# Sodium-glucose co-transporter 2 inhibitor use in patients with diabetes mellitus undergoing endovascular therapy for symptomatic peripheral artery disease

**DOI:** 10.1186/s12933-023-01992-4

**Published:** 2023-10-05

**Authors:** Mitsuyoshi Takahara, Yoshimitsu Soga, Masahiko Fujihara, Osamu Iida, Daizo Kawasaki

**Affiliations:** 1 Department of Diabetes Care Medicine, Osaka University Graduate School of Medicine, 2-2 Yamadaoka, Suita City, 565-0871 Osaka Japan; 2 Department of Cardiology, Kokura Memorial Hospital, 3-2-1 Asano, Kokurakita-ku, Kitakyushu City, 802-0001 Japan; 3 Department of Cardiology, Kishiwada Tokushukai Hospital, 4-27-1, Kamoricho, Kishiwada City, 596-8522 Osaka Japan; 4 Cardiovascular Center, Kansai Rosai Hospital, 3-1-69 Inabaso, Amagasaki City, 660-8511 Hyogo Japan; 5 Cardiovascular Division, Morinomiya Hospital, 2-1-88,Morinomiya, Joto-ku, Osaka City, 536-0025 Japan

**Keywords:** Peripheral artery disease, Diabetes mellitus, Sodium-glucose co-transporter 2 inhibitor, Restenosis

## Abstract

**Background:**

This study aimed to reveal the prevalence of sodium-glucose co-transporter 2 (SGLT2) inhibitor treatment and its association with restenosis risk in patients with diabetes mellitus undergoing endovascular therapy for symptomatic peripheral artery disease.

**Methods:**

We used the database of a multicenter prospective study registering patients with symptomatic peripheral artery disease undergoing femoropopliteal drug-coated balloon treatment in Japan. The current analysis included 1058 patients with diabetes mellitus free from end-stage renal disease. The association of clinical characteristics with SGLT2 inhibitor use was investigated using the logistic regression model. The propensity score matching was adopted to compare the primary patency, i.e., freedom from restenosis, after endovascular therapy between patients treated with and without a SGLT2 inhibitor.

**Results:**

The proportion of SGLT2 inhibitor treatment at revascularization was 14.8% (95% confidence interval, 12.8–17.1%). Younger age, increased body mass index, and increased hemoglobin A1c levels were independently associated with SGLT2 inhibitor use (all P < 0.05). The proportion of SGLT2 inhibitor reached 38.2% (95% confidence interval, 25.4–52.3%) in patients with the three associated factors. The propensity score-matching analysis demonstrated that primary patency was not different between patients treated with a SGLT2 inhibitor and those without it (72.0% [95% confidence interval, 64.1–80.9%] versus 67.8% [62.7–73.3%] at 2 years; P = 0.45).

**Conclusions:**

SGLT2 inhibitors were not rarely used in patients with diabetes mellitus who underwent femoropopliteal endovascular therapy using a drug coated balloon for symptomatic peripheral artery disease in real-world settings. SGLT2 inhibitor treatment was not associated with an increased risk of restenosis.

## Background

Clinical trials have proved that sodium-glucose co-transporter 2 (SGLT2) inhibitors have a protective effect against cardiovascular events [1–4], and more and more patients with diabetes mellitus are treated with the medication. However, at the same time, there is a concern about an increased risk of lower extremity amputation, and care is warranted in the medication use especially in a subpopulation at risk for amputation [2]. Although the concern is still controversial [5–12], decreased lower extremity tissue perfusion related to volume depletion is speculated as a potential mechanism [13, 14]. The involvement of stimulated vasopressin system, potentially inducing vasoconstriction, platelet aggregation, and atherosclerosis, is also discussed [15–19].

Diabetes mellitus is a major risk factor for peripheral artery disease [20–22], and patients undergoing endovascular therapy for symptomatic peripheral artery disease commonly have diabetes mellitus [23, 24]. It remained unknown how reluctant physicians in the real-world settings were to prescribe SGLT2 inhibitors in this population. It also remained unknown whether the prescription would increase the risk of restenosis, or loss of patency, after endovascular therapy. Restenosis after endovascular therapy is attributed chiefly not only by neointimal hyperplasia but also by elastic recoil and thrombosis. If SGLT2 inhibitors lead to volume depletion, vasoconstriction, platelet aggregation, and atherosclerosis, they might adversely affect the patency after revascularization.

The aim of the current study was to reveal the prevalence of SGLT2 inhibitor use and its association with restenosis risk after femoropopliteal endovascular therapy for symptomatic peripheral artery disease in patients with diabetes mellitus.

## Methods

### Study population

The current study used the clinical database of the PrOsPective multiCenter registry Of dRug-coated ballooN for femoropopliteal disease (POPCORN). The POPCORN is an ongoing prospective multicenter observational study that registered adult patients (aged 20 years or older) undergoing drug coated balloon treatment for femoropopliteal lesions of symptomatic peripheral artery disease (Rutherford category 2 to 5) at 81 cardiovascular centers across Japan. A total of 2507 patients were registered between March 2018 and December 2019, and 5-year follow-ups have been scheduled [25]. The study was conducted in accordance with the Declaration of Helsinki, and was approved by the institutional review boards of the participating centers. Informed consent was obtained from the participants or, if not possible, from their families. The POPCORN registry now completed 2-year follow-ups, and the present study utilized the 2-year database of the registry.

Of the 2507 registered patients, 1072 patients had diabetes mellitus and were free from end-stage renal disease. After excluding 14 patients whose data on SGLT2 inhibitor use were missing, 1058 patients were included in the current study.

In patients with multiple femoropopliteal lesions treated, the first registered lesion was included in the present analysis.

### Definitions

Diabetes mellitus was defined as either (1) having anti-diabetic treatment, (2) fasting plasma glucose levels ≥ 126 mg/dl, (3) casual plasma glucose levels ≥ 200 mg/dl, or (4) hemoglobin A1c levels were ≥ 6.5%, according to the domestic guideline [26]. Body mass index was calculated as the weight in kilograms divided by the height in meters squared. Smoking was judged by whether patients smoked at the onset of the index symptomatic peripheral artery disease. Hypertension was defined as either (1) having anti-hypertensive treatment, (2) systolic blood pressure ≥ 140 mmHg, or (3) diastolic blood pressure was ≥ 90 mmHg [27]. Dyslipidemia was defined as either (1) having anti-hyperlipidemic treatment, (2) low-density lipoprotein cholesterol levels were ≥ 140 mg/dl, (3) high-density lipoprotein cholesterol levels were < 40 mg/dl, or (4) triglyceride levels were ≥ 150 mg/dl [28]. Chronic heart failure was determined when either patients were treated with the disease, patients had a history of hospitalization for the disease, or the left ventricular ejection fraction (LVEF) was 40% or less. Heart failure with reduced ejection fraction (HFrEF) was defined determined when LVEF was 40% or less. Coronary artery disease was defined as a history of myocardial infarction, symptomatic myocardial ischemia, or coronary revascularization, whereas stroke denotes a history of cerebral infarction. Estimated glomerular filtration rate (eGFR) was calculated using the Japanese equation from serum creatinine [29]. Chronic kidney disease was defined as eGFR < 60 ml/min/1.73 m^2^ [30]. Chronic limb-threatening ischemia (CLTI) denotes chronic lower extremity ischemia presenting ischemic rest pain or ischemic tissue loss [21, 31]. The vessel diameter was referred to as the reference vessel diameter, which was angiographically assessed at the healthy sites right distal to the index arterial lesion [32]. Severe calcification was defined as the peripheral arterial calcium scoring system (PACSS) grade 4 [33].

Primary patency denotes freedom from restenosis; restenosis was defined as > 2.4 times of the peak systolic velocity ratio on duplex ultrasound, or > 50% of the arterial diameter based on the angiography [34]. Restenosis was scheduled to be routinely monitored by duplex ultrasound at least annually with a time window of ± 2 months, regardless of the presence of ischemic symptoms. Restenosis was also examined when the recurrence of ischemia was clinically suspected of. Target lesion revascularization was clinically driven and defined as reintervention performed for lesions with > 50% diameter stenosis identified by angiography within ± 5 mm of the target lesion after the documentation of recurrent clinical symptoms [34]. Major amputation was defined as surgical excision of the limb above the ankle. Major adverse limb events were referred to as a composite of target lesion revascularization and major amputation.

### Outcome measures

The present study investigated the proportion of SGLT2 inhibitor use. In comparison between patients with a SGLT2 inhibitor and those without it, the primary outcome measure was primary patency (freedom from restenosis). Secondary outcome measures included freedom from target lesion revascularization, bypass conversion, major amputation, major adverse limb events, all-cause death, and cardiovascular death.

### Statistical analysis

Data on baseline characteristics are presented as the mean ± standard deviation (SD) for continuous variables and the percentage for discrete variables, if not otherwise mentioned. A P value of < 0.05 was considered statistically significant, and 95% confidence intervals were reported where appropriate. All statistical analyses were performed with R version 4.1.1 (R Development Core Team, Vienna, Austria).

We divided the study population into patients treated with a SGLT2 inhibitor at revascularization and those without it. The 95% confidence interval of the proportion of SGLT2 inhibitor use was calculated with the Clopper-Pearson exact method. The differences in baseline characteristics between the groups with and without a SGLT2 inhibitor were crudely tested by the Welch’s *t* test for continuous variables, and by the chi-squared test for discrete variables. The independent association of baseline characteristics with SGLT2 inhibitor use was explored using the multivariable logistic regression model in which variables with a significant crude intergroup difference were entered.

In the groups with and without a SGLT2 inhibitor, the follow-up data after the discontinuation and the start, respectively, of the medication were excluded from the present analysis. When clinical outcomes were compared between the two groups, the propensity score matching was adopted to minimize the inter-group difference in baseline characteristics. The propensity score was developed using the logistic regression model that included the following variables: age, sex, smoking, hypertension, dyslipidemia, chronic heart failure, HFrEF, LVEF, atrial fibrillation, coronary artery disease, stroke, hemoglobin A1c (HbA1c) levels, medications (aspirin, P2Y12 inhibitors, cilostazol, anticoagulants, and statin), CLTI, ankle-brachial index, reference vessel diameter, lesion length, severe calcification, chronic total occlusion, and the type of drug coated balloon. Matching was performed on the logit of the propensity score within the caliper of 0.2 SD of the logit of the propensity score. To maximize the statistical power to detect intergroup prognostic differences, we extracted as many matched samples in the group without a SGLT2 inhibitor to one in the group with it as possible. After matching, the intergroup difference was analyzed with stratification by the pairs, and weighted descriptive statistics are reported. The intergroup balance in baseline characteristics was assessed using the standardized difference. Time-to-events (primary and secondary outcome measures) were estimated by the Kaplan–Meier method and were compared between the groups by the stratified log-rank test. Patients who were treated with an SGLT2 inhibitor at revascularization but thereafter discontinued the medication were censored at the discontinuation. Correspondingly, patients who were not treated with an SGLT2 inhibitor at revascularization but thereafter started the medication were censored at the start. The interaction effect of baseline characteristics on the association of SGLT2 inhibitor use with restenosis risk was analyzed using the Cox proportional hazards regression model stratified by matched pairs.

## Results

**Fig. 1:**
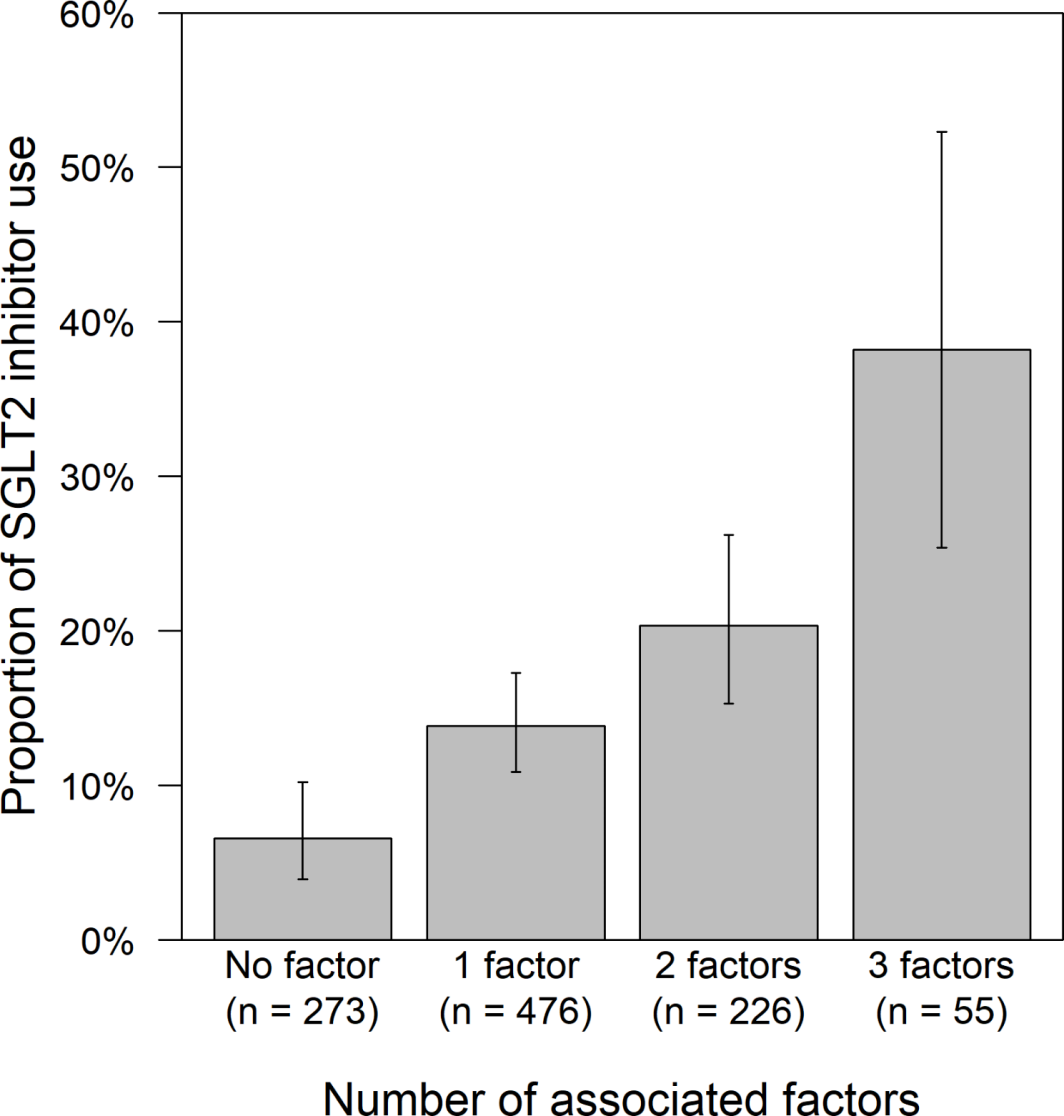
Proportion of SGLT2 inhibitor use by the number of the associated factors. The associated factors are younger age (< 70 years), increased body mass index (≥ 25 kg/m^2^), and increased hemoglobin A1c levels (≥ 7.0% [52 mmol/mol]). Error bars indicate 95% confidence intervals. SGLT2, sodium-glucose co-transporter 2

Of a total of 1058 patients undergoing femoropopliteal endovascular therapy with drug coated balloon for symptomatic peripheral artery disease, 157 patients (14.8%) were treated with a SGLT2 inhibitor. The 95% confidence interval of the proportion was calculated to be 12.8–17.1%. The remaining 901 patients were not treated with a SGLT2 inhibitor at revascularization. The baseline characteristics of patients treated with and without a SGLT2 inhibitor are summarized in Table 1. Patients treated with a SGLT2 inhibitor had (1) a younger age (72 ± 9 versus 75 ± 8 years; P < 0.001), (2) a higher body mass index (24.2 ± 3.7 versus 23.1 ± 3.5 years; P = 0.002), a higher prevalence of (3) dyslipidemia (96.2% versus 88.9%; P = 0.008) and (4) coronary artery disease (65.4% versus 56.0%; P = 0.037), (5) higher HbA1c levels (7.7 ± 1.4% versus 7.2 ± 1.2% [61 ± 15 versus 55 ± 13 mmol/mol]; P < 0.001), and (6) a higher proportion of statin use (83.1% versus 70.3%; P = 0.001) than patients treated without a SGLT2 inhibitor. The multivariable logistic regression model, including those six factors with a significant intergroup difference, demonstrated that age, body mass index, and HbA1c levels were independently associated with SGLT2 inhibitor use (Table 2). The adjusted odds ratios were 0.80 (95% confidence interval, 0.65–0.99; P = 0.042) per 10-year increase, 1.40 (1.08–1.78; P = 0.010) per 5 kg/m^2^ increase, and 1.31 (1.14–1.49; P < 0.001) per 1% (10.93 mmol/mol) increase, respectively. The use of SGLT2 inhibitors was more commonly seen in patients with the accumulation of these associated factors (Fig. 1); the proportion reached 38.2% (95% confidence interval, 25.4–52.3%) in patients with younger age (< 70 years), increased body mass index (≥ 25 kg/m^2^) and increased HbA1c levels (≥ 7.0% [≥ 52 mmol/mol]).

**Table 1:** Baseline characteristics of the study population

	Patients with a SGLT2 inhibitor	Patients without a SGLT2 inhibitor	P value
	(n = 157)	(n = 901)	
Age (years)	72 ± 9	75 ± 8	< 0.001
Male sex	66.2%	69.4%	0.49
Body mass index (kg/m^2^)	24.2 ± 3.7	23.1 ± 3.5	0.002
Smoking	25.5%	23.8%	0.71
Hypertension	87.3%	85.0%	0.54
Dyslipidemia	96.2%	88.9%	0.008
Chronic kidney disease	56.1%	56.9%	0.90
eGFR (ml/min/1.73 m^2^)	60 ± 22	58 ± 22	0.28
Chronic heart failure	17.2%	14.4%	0.44
HFrEF	3.8%	2.9%	0.70
(missing data)	0.6%	0.6%	> 0.99
LVEF (%)	61 ± 11	62 ± 10	0.34
(missing data)	12.1%	7.9%	0.11
Atrial fibrillation	10.8%	12.1%	0.75
Coronary artery disease	65.4%	56.0%	0.037
(missing data)	0.6%	0.0%	0.32
Stroke	14.0%	18.6%	0.20
HbA1c (%)	7.7 ± 1.4	7.2 ± 1.2	< 0.001
IFCC units (mmol/mol)	61 ± 15	55 ± 13	< 0.001
(missing data)	3.8%	2.4%	0.47
SGLT2 inhibitor use			–
Empagliflozin	35.0%	–	
Dapagliflozin	20.4%	–	
Canagliflozin	15.3%	–	
Other SGLT2 inhibitors	29.3%	–	
Aspirin use	80.8%	81.7%	0.88
(missing data)	0.6%	0.1%	0.69
P2Y12 inhibitor use	87.3%	86.6%	0.93
(missing data)	0.0%	0.3%	> 0.99
Cilostazol use	29.2%	23.0%	0.12
(missing data)	1.9%	1.0%	0.56
Anticoagulant use	20.0%	17.4%	0.51
(missing data)	1.3%	0.1%	0.086
Statin use	83.1%	70.3%	0.001
(missing data)	1.9%	0.7%	0.27
CLTI	25.5%	23.1%	0.58
Ankle-brachial index	0.58 ± 0.24	0.62 ± 0.22	0.051
(missing data)	1.9%	1.3%	0.84
Vessel diameter (mm)	4.9 ± 1.0	4.8 ± 0.9	0.33
(missing data)	1.3%	0.4%	0.48
Lesion length (cm)	14.4 ± 9.3	13.8 ± 9.7	0.48
(missing data)	0.6%	0.0%	0.32
Severe calcification	10.2%	10.3%	> 0.99
(missing data)	0.0%	0.1%	> 0.99
Chronic total occlusion	33.5%	28.3%	0.22
(missing data)	1.3%	0.0%	0.017
Drug coated balloon use			0.70
IN.PACT Admiral	77.1%	78.8%	
Lutonix	22.9%	21.2%	

Data are percentages for discrete variables, and means ± standard deviations for continuous variables. CLTI, chronic limb-threatening ischemia; eGFR, estimated glomerular filtration rate; HbA1c, hemoglobin A1c; HFrEF, heart failure with reduced ejection fraction; IFCC, the International Federation of Clinical Chemistry and Laboratory Medicine; LVEF, left ventricular ejection fraction; SGLT2, sodium-glucose co-transporter 2

**Table 2:** Association of baseline characteristics with SGLT2 inhibitor use

	Adjusted odds ratio
Age (per 10-year increase)	0.80 [0.65 to 0.99] (P = 0.042)
Body mass index (per 5 kg/m^2^ increase)	1.40 [1.09 to 1.79] (P = 0.008)
Dyslipidemia	1.76 [0.63 to 4.95] (P = 0.28)
Coronary artery disease	1.39 [0.94 to 2.04] (P = 0.097)
HbA1c (per 1% [10.93-mmol/mol] increase)	1.31 [1.14 to 1.49] (P < 0.001)
Statin use	1.55 [0.91 to 2.65] (P = 0.11)

Data are adjusted odds ratios [95% confidence intervals] (P values), derived from the multivariable logistic regression model including all the variables in the table, which were significantly different between patients treated with and without a SGLT2 inhibitor (see Table 1). HbA1c, hemoglobin A1c; SGLT2, sodium-glucose co-transporter 2

**Fig. 2:**
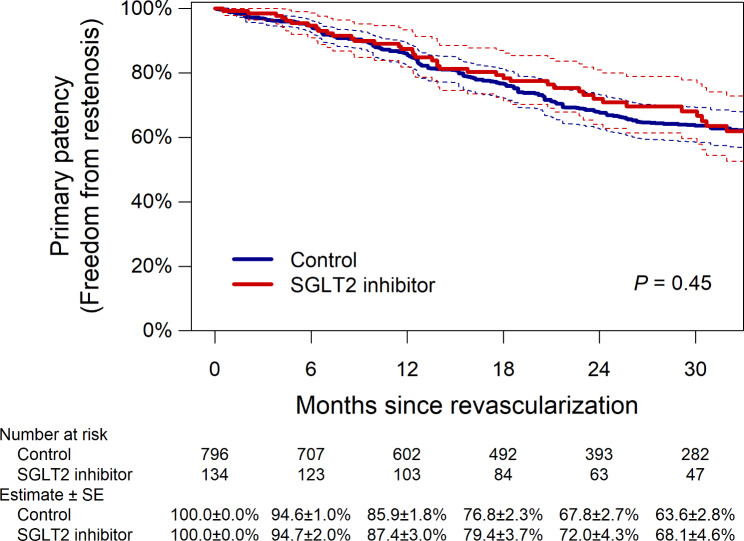
Primary patency (freedom from restenosis) in the propensity score-matched population. Dotted lines indicate 95% confidence intervals. SE, standard error; SGLT2, sodium-glucose co-transporter 2

**Fig. 3:**
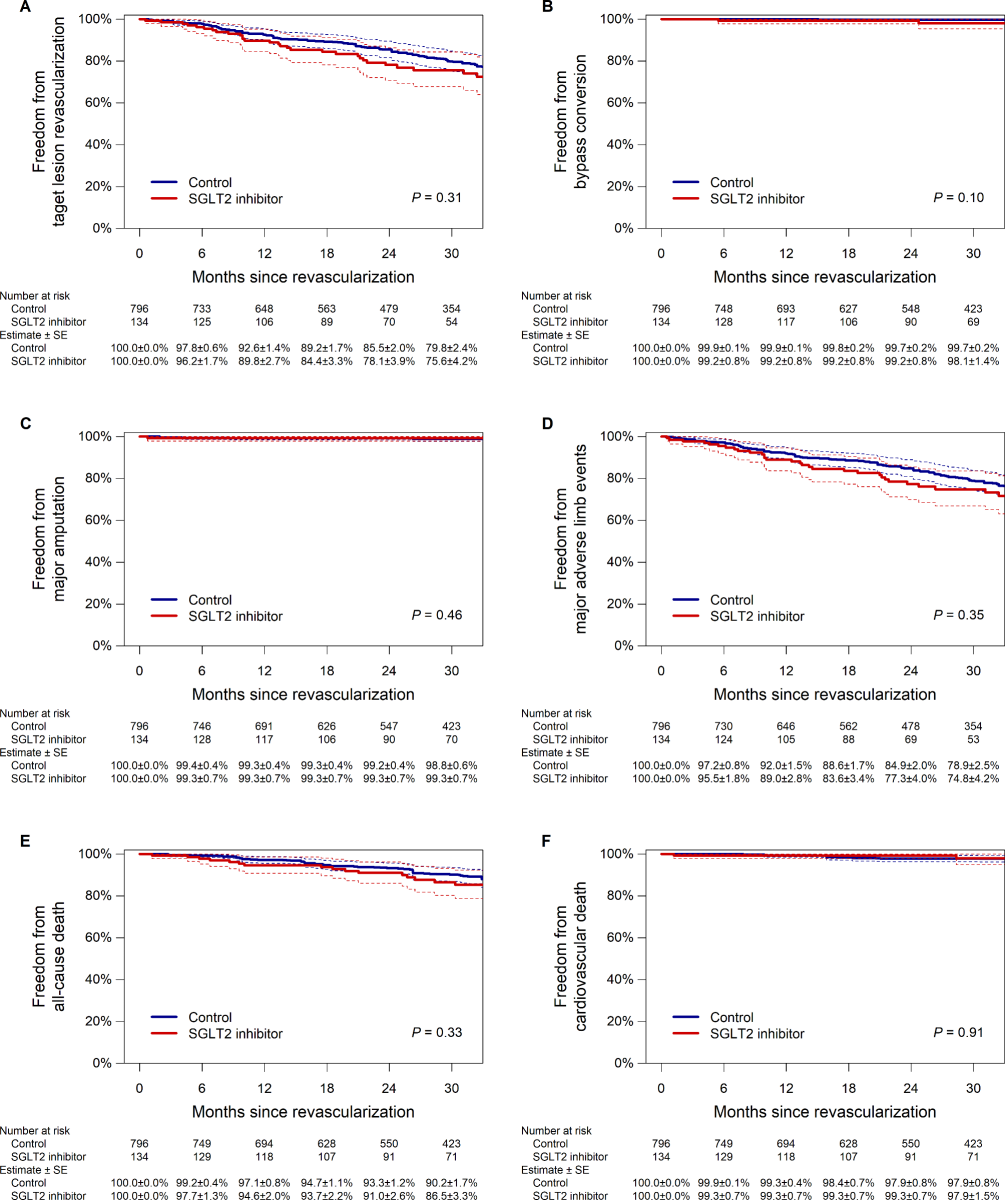
Freedom from target lesion revascularization (**A**), bypass conversion (**B**), major amputation (**C**), major adverse limb events (**D**), all-cause death (**E**), and cardiovascular death (**F**) in the propensity score-matched population. Dotted lines indicate 95% confidence intervals. SE, standard error; SGLT2, sodium-glucose co-transporter 2

**Fig. 4:**
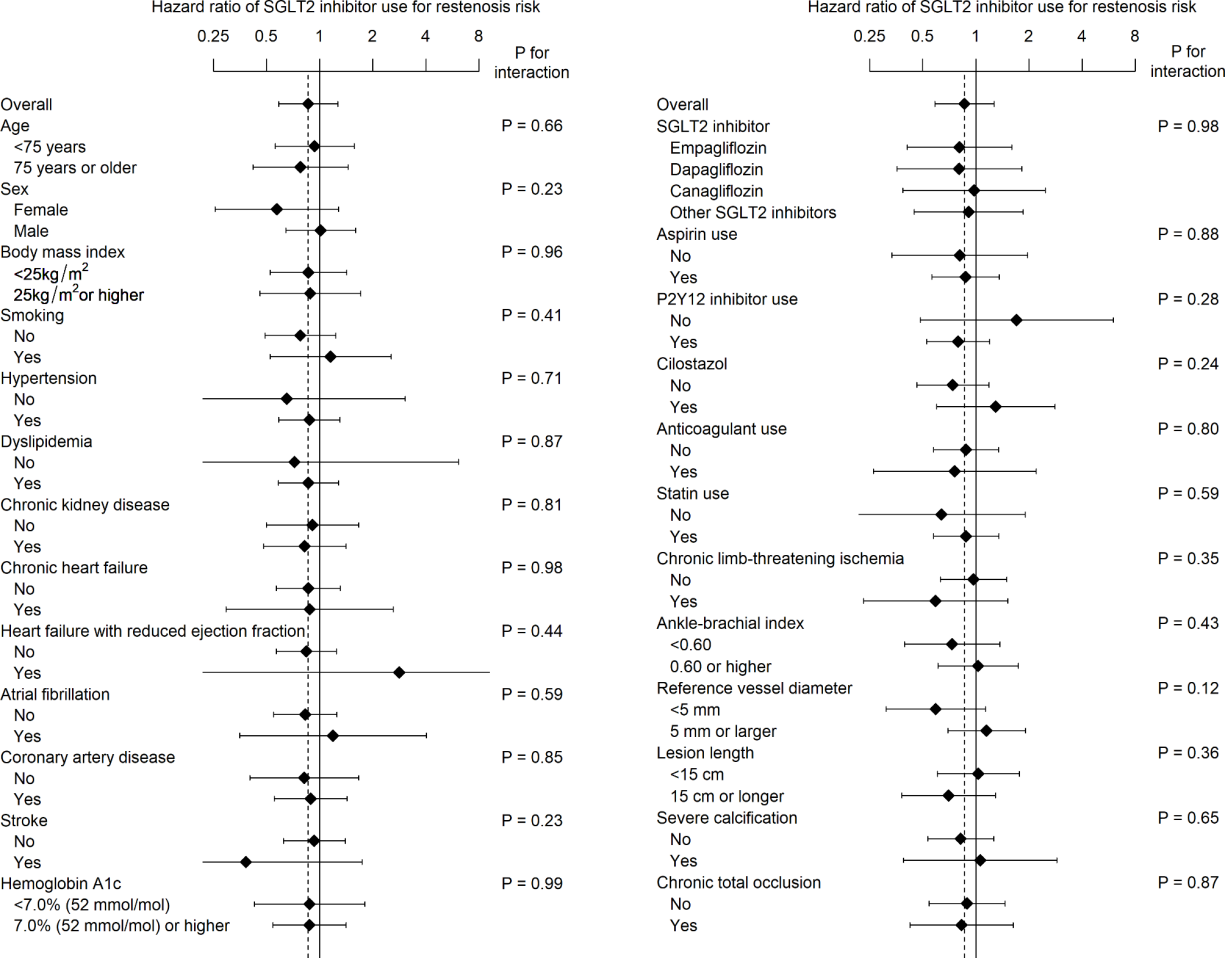
Interaction effect of baseline characteristics on the association of SGLT2 inhibitor use with restenosis risk in the propensity score-matched population. Error bars indicate 95% confidence intervals. SGLT2, sodium-glucose co-transporter 2

Of the 157 patients treated with an SGLT2 inhibitor at revascularization, 5 patients (2.6%) discontinued the medication during follow-up (2 patients discontinued it within the first week, and the remaining 3 discontinued it more than 1 year after revascularization). Of the 901 patients who were not treated with an SGLT2 inhibitor at revascularization, 12 patients (1.1%) thereafter started the medication (1 patient started it within the first week, 2 patients started it between 1 and 3 months, 5 patients started it between 3 and 6 months, 3 patients started it between 6 and 12 months, and 1 patient started it more than 1 year after revascularization). During a median follow-up period of 23.5 (interquartile range, 12.0–35.6) months, restenosis, or loss of primary patency, was observed in 332 patients. The propensity score matching extracted 134 pairs (134 patients treated with a SGLT2 inhibitor and 796 patients without it), with no remarkable intergroup difference in baseline characteristics (Table 3). As illustrated in Fig. 2, the primary patency (i.e., freedom from restenosis) was not significantly different between the groups (P = 0.45); the 1-year rate of primary patency was 87.4% (95% confidence interval, 81.8–93.4%) in patients treated with a SGLT2 inhibitor and 85.9% (82.5–89.4%) in those without it, respectively, whereas the 2-year corresponding rate was 72.0% (64.1–80.9%) and 67.8% (62.7–73.3%). No significant intergroup difference was found in secondary outcome measures (all P > 0.05) (Fig. 3). The interaction effect of baseline characteristics on the association of SGLT2 inhibitor use with restenosis risk is shown in Fig. 4. No baseline characteristics had a significant interaction effect on the association.

**Table 3:** Baseline characteristics of the propensity score-matched population

	Patients with a SGLT2 inhibitor	Patients without a SGLT2 inhibitor	Standardized difference
	(n = 134)	(n = 796)	
Age (years)	72 ± 9	73 ± 8	7.1
Male sex	67.2%	68.0%	1.8
Body mass index (kg/m^2^)	24.3 ± 3.7	24.3 ± 3.8	1.5
Smoking	26.1%	28.5%	5.4
Hypertension	88.8%	88.2%	1.9
Dyslipidemia	96.3%	96.0%	1.3
Chronic kidney disease	56.0%	54.1%	3.8
eGFR (ml/min/1.73 m^2^)	60 ± 21	60 ± 22	2.4
Chronic heart failure	19.4%	19.1%	0.9
HFrEF	4.5%	4.3%	1.1
(missing data)	0.7%	0.7%	0.8
LVEF (%)	61 ± 12	61 ± 11	4.8
(missing data)	10.4%	10.9%	1.6
Atrial fibrillation	11.2%	12.5%	4.1
Coronary artery disease	68.7%	68.6%	0.2
(missing data)	0.0%	0.0%	0.0
Stroke	14.9%	15.8%	2.4
HbA1c (%)	7.7 ± 1.3	7.7 ± 1.6	0.4
IFCC units (mmol/mol)	61 ± 14	61 ± 17	0.4
(missing data)	0.0%	0.0%	0.0
SGLT2 inhibitor use			
Empagliflozin	37.3%	–	–
Dapagliflozin	18.7%	–	–
Canagliflozin	14.9%	–	–
Other SGLT2 inhibitors	29.1%	–	–
Aspirin use	80.6%	79.5%	2.7
(missing data)	0.0%	0.0%	0.0
P2Y12 inhibitor use	88.1%	88.9%	2.6
(missing data)	0.0%	0.0%	0.0
Cilostazol use	28.8%	28.5%	0.5
(missing data)	1.5%	1.3%	1.4
Anticoagulant use	20.1%	21.2%	2.5
(missing data)	0.0%	0.0%	0.0
Statin use	84.2%	83.2%	2.7
(missing data)	0.7%	1.3%	5.3
CLTI	25.4%	27.9%	5.8
Ankle-brachial index	0.58 ± 0.24	0.58 ± 0.24	2.0
(missing data)	1.5%	1.4%	1.1
Vessel diameter (mm)	5.0 ± 0.9	5.0 ± 0.9	0.4
(missing data)	0.0%	0.0%	0.0
Lesion length (cm)	14.6 ± 9.4	14.3 ± 10.1	3.3
(missing data)	0.0%	0.0%	0.0
Severe calcification	9.7%	10.0%	1.1
(missing data)	0.0%	0.0%	0.0
Chronic total occlusion	33.6%	31.1%	5.4
(missing data)	0.0%	0.0%	0.0
Drug coated balloon use			
IN.PACT Admiral	76.9%	76.6%	0.6
Lutonix	23.1%	23.4%	0.6

Data are weighted percentages for discrete variables, and weighted means ± weighted standard deviations for continuous variables. CLTI, chronic limb-threatening ischemia; eGFR, estimated glomerular filtration rate; HbA1c, hemoglobin A1c; HFrEF, heart failure with reduced ejection fraction; IFCC, the International Federation of Clinical Chemistry and Laboratory Medicine; LVEF, left ventricular ejection fraction; SGLT2, sodium-glucose co-transporter 2

## Discussion

SGLT2 inhibitors were not rarely used in patients with diabetes mellitus who underwent femoropopliteal endovascular therapy using a drug coated balloon for symptomatic peripheral artery disease in Japan. The proportion of SGLT2 inhibitor treatment at revascularization was 14.8% (95% confidence interval, 12.8–17.1%) in the overall population. SGLT2 inhibitors were more frequently used in patients with younger age, increased body mass index, and increased HbA1c levels. The proportion reached 38.2% (95% confidence interval, 25.4–52.3%) in patients accumulating the three clinical features. The subsequent propensity score-matching analysis demonstrated that primary patency was not different between patients treated with a SGLT2 inhibitor and those without it. To the best of our knowledge, this is the first report on SGLT2 inhibitor use in patients undergoing revascularization for symptomatic peripheral artery disease.

In the Canagliflozin Cardiovascular Assessment Study (CANVAS) [2], patients treated with canagliflozin had a greater risk of amputation of toes, feet, or legs than those with placebo. The finding raised clinical concerns about SGLT2 inhibitor use in patients at risk for amputation. Although the issue remained controversial [5–12], the European Medicines Agency (EMA) and the Drug Safety Communications of the US Food and Drug Administration (FDA) promptly issued the relevant warning in 2017 [35, 36]. It is no doubt that the result of the CANVAS made a strong impact on clinical practice. On the other hand, protective effects of SGLT2 inhibitors against cardiovascular events [1–4] were clinically attractive, especially in patients at risk of those adverse events. Peripheral artery disease often coexists with other cardiovascular diseases [20, 21]; in this context, physicians could regard patients with peripheral artery disease as candidates for beneficiaries of the protective effects against cardiovascular events. The present multicenter study, registering patients between March 2018 and December 2019, i.e., after the publishment of the potential amputation risk, can show the real-world trends in the prescription of SGLT2 inhibitors in patients with diabetes mellitus undergoing endovascular therapy for symptomatic peripheral artery disease.

In the present study, 14.8% (95% confidence interval, 12.8–17.1%) were treated with a SGLT2 inhibitor at revascularization. Observational studies of outpatients with diabetes mellitus at clinics and hospitals in Japan reported the proportion of SGLT2 inhibitor use was ranged from 16.1 to 23.6% [37, 38]. Compared with those reports, the corresponding proportion in the present study seemed slightly lower, but not extremely low. The reluctance of physicians to administer SGLT2 inhibitors in patients requiring revascularization for symptomatic peripheral artery disease would be limited in real-world settings.

Younger age, increased body mass index, and increased HbA1c levels were independently associated with SGLT2 inhibitor use. The associations were consistent with previous reports on a general population with diabetes mellitus [38]. Lower prescription rates in older patients would possibly reflect clinical concerns relating to potential adverse effects and insufficient safety data in the population [39]. Patients with increased body mass index were more likely to be treated with a SGLT2 inhibitor, probably because its pharmacological effect, i.e., urinary glucose excretion, accompanied not only by reduced blood glucose levels but also by reduced body weight [40], would be assumed by physicians to be suitable for patients with obesity. On the other hand, the positive association of HbA1c levels and SGLT2 inhibitor use would reflect the domestic trends, in which SGLT2 inhibitors were still likely to be prescribed as the second and subsequent line therapy, rather than the first line therapy in Japan [41]; SGLT2 inhibitors might be more likely to be selectively administered to patients whose glycemic control was considerably poor.

After the result of the CANVAS was published [2], various speculations were made about potential mechanisms of a possibly increased amputation risk. Some hypothesized that the diuretic effect of SGLT2 inhibitors might induce volume depletion that might decrease lower extremity tissue perfusion [13, 14]. A potential involvement of stimulated vasopressin system was also speculated [14–19, 42–48]. The vasopressin receptor V1a subtype is expressed in vascular smooth muscle cells and platelet membrane, through which vasopressin exercise a vasoconstricting effect in lower extremity arteries, and induces platelet aggregation [16–18], while the V2 receptor is expressed in endothelium and regulates circulating levels of coagulation factor VIII, von Willebrand factor, and tissue plasminogen activator [16, 49, 50]. The stimulation of the vasopressin system by SGLT2 inhibitor treatment would potentially induce vasoconstriction and thrombogenesis, and progress atherosclerosis.

On the other hand, it has been pointed out that the diuresis of SGLT2 inhibitors after the early phase of the treatment may be if any small [13, 51]. Furthermore, the link of vasopressin with cardiovascular events could not explain the protective effect of SGLT2 inhibitors against cardiovascular events which has been proved by a series of clinical trials [1–4]. The effect of SGLT2 inhibitors on volume depletion and vasopressin stimulation would be not so large enough to affect clinically manifested atherosclerosis-related outcomes. The present study found that patients treated with a SGLT2 inhibitor did not have a significantly higher restenosis risk than those without it. The adverse effect of SGLT2 inhibitors would be clinically ignorable, at least on the patency after endovascular therapy using a drug coated balloon. The present study did not find a significant risk reduction of all-cause and cardiovascular death by SGLT2 inhibitor use, which might come from a relatively small number of observed events.

The present study extracted data from the POPCORN study, being the largest prospective registry on femoropopliteal endovascular therapy using a drug coated balloon in Japan [25]. The study was practically an all comers registry conducted in clinical practice, and we believe the registry could demonstrate the real-world data in Japan. It was reported that the presence of diabetes did not affect the primary patency after femoropopliteal endovascular therapy using a drug coated balloon [25, 52]. The 1-year rate of primary patency in the present study was 87.4% (81.8–93.4%) and 85.9% (82.5–89.4%) in patients with and without SGLT2 inhibitor use, respectively. The result was comparable to that reported by other real-world studies, ranging from 80 to 90% [52–55], which would partially support the representativeness and generalizability of our data as real-world ones.

Our study has several limitations. First, the current registry did not collect data on the patients’ adherence to treatment including medication, or the reason why attending physicians administered or did not administer SGLT2 inhibitors to the patients. They might affect the results. Neither did we collect data on whether, in patients treated with a SGLT2 inhibitor at revascularization, the medication was newly started after the referral to the vascular center, or was already prescribed at the previous clinic and was continued as it was. It also remained unknown whether, in patients without a SGLT2 inhibitor at revascularization, the medication was prescribed at the previous clinic but was discontinued after the referral, or was not prescribed ever. Reasons for administering anticoagulants were also unknown. Low-dose rivaroxaban was not approved or reimbursed at that time in Japan, and anticoagulants were clinically administered to patients with atrial fibrillation, prosthetic valve replacement, venous thrombosis, and other thromboembolisms. However, we did not collect data on for what reason patients were treated with an anticoagulant. Second, the association between clinical characteristics and SGLT2 inhibitor use was analyzed cross-sectionally, and therefore we were unable to prove their causal relationships. Third, the current study did not survey detailed diabetes-related information that might affect SGLT2 inhibitor prescription, including duration of diabetes mellitus, insulin resistance, and beta-cell function. Fourth, the current analysis only included patients who underwent endovascular therapy with a drug coated balloon for femoropopliteal artery disease. It remains unknown whether similar findings were observed in patients undergoing other revascularization strategies with other devices or for other vascular territories. Fifth, the present study analyzed the 2-year database, and longer-term clinical outcomes remained to be revealed. Primary patency of femoropopliteal endovascular devices is often assessed primarily at 1 year [56–58]. The present study, analyzing a 2-year database, covered this main follow-up period. However, far longer-term clinical outcomes remained unknown. Sixth, no data were available on incident minor amputation or incident hospitalization due to heart failure. Finally, the current study was conducted in Japan. The prescription pattern would be influenced by the domestic healthcare system and ethic difference, and would vary from country to country. Future studies in other countries are necessary to validate the current findings.

## Conclusions

SGLT2 inhibitors were not rarely used in patients with diabetes mellitus who underwent femoropopliteal endovascular therapy using a drug coated balloon for symptomatic peripheral artery disease in Japan. Primary patency was not different between patients treated with a SGLT2 inhibitor and those without it.

## Data Availability

The data that support the findings of this study are not publicly available due to ethical reasons but are available from the corresponding author upon reasonable request and with permission of the ethics committee of the participating institutions.

## List of abbreviations

CANVAS: Canagliflozin Cardiovascular Assessment Study
CLTI: chronic limb threatening ischemia
eGFR: estimated glomerular filtration rate
EMA: European Medicines Agency
FDA: Food and Drug Administration
HbA1c: hemoglobin A1c
HFrEF: heart failure with reduced ejection fraction
IFCC: the International Federation of Clinical Chemistry and Laboratory Medicine
LVEF: left ventricular ejection fraction
PACSS: peripheral arterial calcium scoring system
POPCORN: prospective multicenter registry ff drug-coated balloon for femoropopliteal disease
SD: standard deviation
SGLT2: sodium-glucose co-transporter 2
